# Trends in survival of ovarian clear cell carcinoma patients from 2000 to 2015

**DOI:** 10.3389/fonc.2024.1360663

**Published:** 2024-03-07

**Authors:** Bing-Qian Tian, Shu-Wen Wang, Jing-Ying Xu, San-Gang Wu, Juan Zhou

**Affiliations:** ^1^ School of Medicine, Xiamen University, Xiamen, China; ^2^ Department of Obstetrics and Gynecology, The First Affiliated Hospital of Xiamen University, School of Medicine, Xiamen University, Xiamen, China; ^3^ Department of Radiation Oncology, Xiamen Cancer Center, Xiamen Key Laboratory of Radiation Oncology, The First Affiliated Hospital of Xiamen University, School of Medicine, Xiamen University, Xiamen, China

**Keywords:** ovarian cancer, survival trend, clear cell carcinoma, annual percentage change, SEER

## Abstract

**Purpose:**

To analyze changes in survival outcomes in patients with ovarian clear cell carcinoma (OCCC) treated consecutively over a 16-year period using a population-based cohort.

**Methods:**

We conducted a retrospective analysis of OCCC from 2000 to 2015 using data from the Surveillance, Epidemiology, and End Results (SEER) program. The ovarian cancer-specific survival (OCSS) and overall survival (OS) were analyzed according to the year of diagnosis. Joinpoint Regression Program, Kaplan-Meier analysis, and multivariate Cox regression analyses were used for statistical analysis.

**Results:**

We included 4257 patients in the analysis. The analysis of annual percentage change in OCSS (P=0.014) and OS (P=0.006) showed that patients diagnosed in later years had significantly better outcomes compared to those diagnosed in early years. The results of the multivariate Cox regression analyses showed that the year of diagnosis was the independent prognostic factor associated with OCSS (P=0.004) and had a borderline effect on OS (P=0.060). Regarding the SEER staging, the OCSS (P=0.017) and OS (P=0.004) of patients with distant stage showed a significant trend toward increased, while no significant trends were found in the survival of patients with localized or regional stage diseases. Similar trends were found in those aged <65 years or those treated with surgery and chemotherapy. However, no statistically significant changes in the survival rate were found in those aged ≥65 years or those receiving surgery alone regardless of SEER stage during the study period.

**Conclusions:**

Our study observed a significant increase in the survival outcomes in OCCC from 2000 to 2015, and patients aged <65 years and those with distant stage experienced a greater improvement in survival.

## Introduction

Epithelial ovarian cancer (EOC) is the gynecological tumor with the highest mortality rate (1). Due to the relatively insidious onset of this disease, approximately 70% of patients were diagnosed with advanced-stage disease (1). BRCA1/2 germline mutations are the strongest known genetic risk factors for EOCs and are found in 6-15% of women diagnosed with that disease. BRCA1/2 carriers with EOCs respond better than non-carriers to platinum-based chemotherapies. This yields greater survival, even though the disease is generally diagnosed at a later stage and higher grade (2). According to the WHO classification of tumors, there are five main histological subtypes of EOC, including high-grade serous, low-grade serous, mucinous, endometrioid, and clear cell carcinoma of the ovary (3). Another rare and highly aggressive type of EOC is ovarian carcinosarcoma, which accounts for less than 5% of ovarian cancer (3). Each of the identified histotypes has distinct clinicopathological and molecular features, and different developmental origins (4). Due to the complexity of histological classification, there are significant differences in the availability and accessibility of treatment options for each subtype, resulting in varying patient outcomes (5). Phosphatidylinositol-4,5-bisphosphate 3-kinase (PI3K) pathway is frequently upregulated in EOC and plays an important role in chemoresistance and preservation of genomic stability, as it is implicated in many processes of DNA replication and cell cycle regulation. The inhibition of the PI3K may lead to genomic instability and mitotic catastrophe through a decrease of the activity of the spindle assembly checkpoint protein Aurora kinase B and consequently increase the occurrence of lagging chromosomes during prometaphase (6).

Ovarian clear cell carcinoma (OCCC) is a rare and unique malignancy of the EOC and has an incidence of 0.6/100,000 (1). The incidence of OCCC in East Asian populations has been increasing, accounting for nearly 30% of EOC (7), while OCCC only accounts for 5-10% in the United States (US) population (8), suggesting that there may be some geographical and ethnic variation in the incidence of OCCC. OCCC is characterized by the presence of clear cells with a hobnail appearance and is often associated with endometriosis (9–11). Moreover, OCCC is known to have distinct clinicopathologic features, genetic alterations, and prognosis compared to other subtypes of EOC (5). OCCC has a unique genetic profile with a lower p53 mutation rate and a lower BRCA1/2 mutation rate but higher mutation rates in AT-rich interaction domain 1A (ARID1A), PIK3CA, and PTEN compared to high-grade serous EOC (12).

Generally, the overall survival (OS) rates for advanced OCCC have been reported to be lower compared to other histological subtypes of EOC (13–15). Despite a lower rate of responses due to intrinsic chemoresistance, the treatment strategy for OCCC is the same used for high-grade serous EOC, which includes aggressive cytoreductive surgery and platinum-based adjuvant chemotherapy. Over the past few decades, there have been significant efforts to improve early detection and develop targeted therapies for EOC (5). Several biological agents have been investigated in patients with newly diagnosed, persistent, or recurrent OCCC, and bevacizumab combined with platinum-taxane chemotherapy had a response rate of 63.6% and one-year progression-free survival was 50.5%, suggesting that the addition of bevacizumab to chemotherapy for OCCC could be an important treatment strategy (16, 17). The response rate in those treated with bevacizumab was higher than other biological agents and bevacizumab was approved for the treatment of EOC starting in 2007 (17–19). Survival trends are crucial in assessing the effectiveness of treatment strategies and advancements in medical care for OCCC. However, it is still unclear whether the advancement of treatment strategies will bring survival improvement to OCCC. This study aimed to investigate the changes in ovarian cancer-specific survival (OCSS) and OS of OCCC patients treated consecutively over a 16-year period using a population-based cohort.

## Materials and methods

### Patients

**Figure 1:**
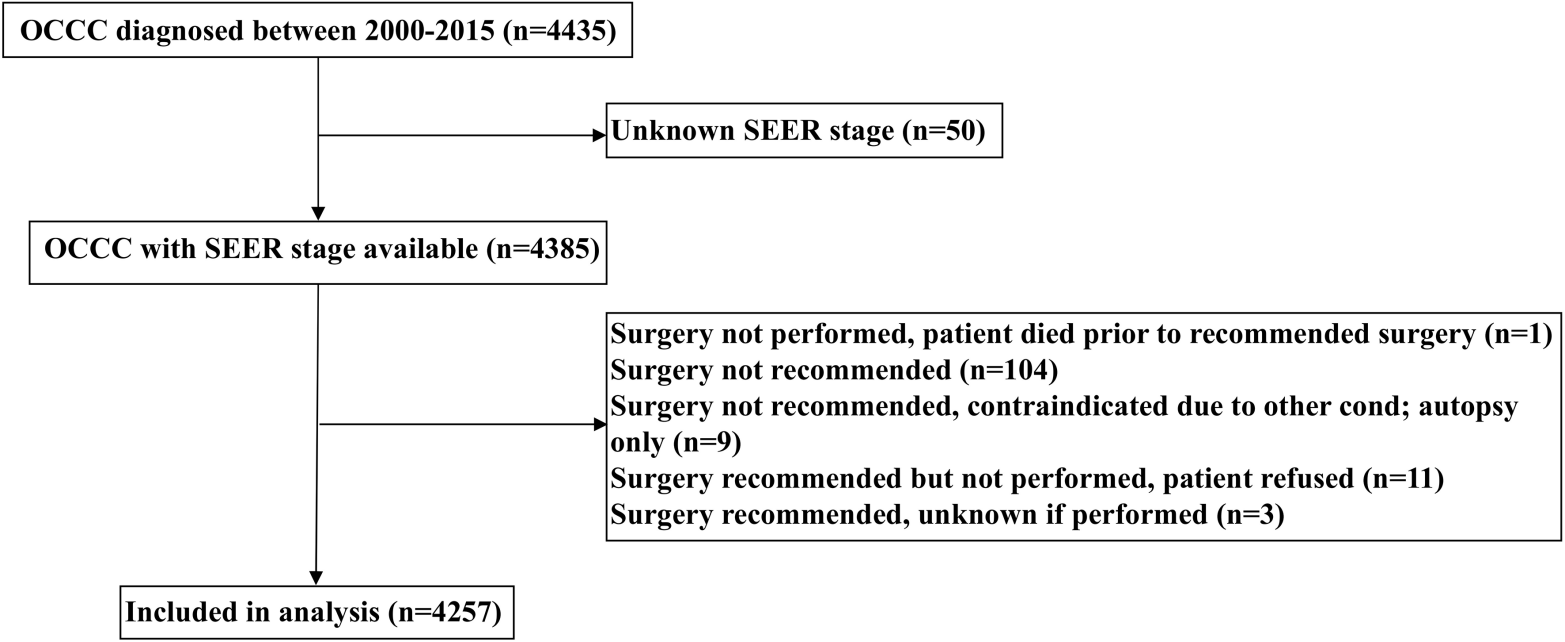
The flowchart of the cohort selection.

Patients diagnosed with OCCC between 2000 and 2015 were included retrospectively from the Surveillance, Epidemiology, and End Results (SEER) database (20). We identified patients who met the following inclusion criteria: 1) diagnosed with OCCC (International Classification of Diseases for Oncology, 3rd ed. [ICD-O-3], primary site: C56.9-ovary) (ICD-O-3 codes 8290/3, 8310/3, 8313/3, 8443/3, and 8444/3); 2) available SEER staging; 3) received surgery with or without chemotherapy. The patient selection flowchart has listed in **Figure 1**. We excluded patients with non-positive pathological diagnoses in this study. Institutional review board approval was not required for our study as the SEER database contains de-identified information.

### Variables

We included the following variables in the analysis: year of diagnosis, age, race, tumor grade, SEER stage, CA125 status, and treatment receipt. The classification of the years of diagnosis was 2000-2007 and 2008-2015, which was due to the approval of bevacizumab for the treatment of EOC starting in 2007 (18, 19). SEER stage is defined by the derived SEER Summary Stage 2000 variable (21). It utilizes the Collaborative Staging algorithm to merge clinical and pathologic information regarding the extent of disease and assign a stage for diagnoses made in 2004 and beyond. The SEER staging system corresponds to the commonly used International Federation of Gynecology and Obstetrics (FIGO) staging system in the following way: localized (FIGO I-A, I-B, I-not otherwise specified [NOS]), regional (FIGO I-C, II-A, II-B, II-C, II-NOS), and distant stage (FIGO III-A, III-B, III-C, III-NOS, IV) (21). Elevated CA125 was defined as the level of CA125 >35 ug/ml. The primary outcomes of this study were OCSS and OS. OCSS was defined as the time period from the diagnosis of OCCC to death specifically caused by ovarian cancer. OS was defined as the duration from the diagnosis of OCCC to death from any cause.

### Statistical analysis

Statistical significances in categorical variables by year of diagnosis were compared using chi-square tests. We utilized the Joinpoint Regression Program, version 4.9.1.0 (National Cancer Institute) to analyze the time trends in survival outcomes. We also explored the impact of variables such as age at diagnosis, SEER staging, and treatment receipt on changes in patient survival, and the annual percentage change (APC) metric was chosen to describe the average percentage change in survival in a given period for one year relative to survival in the previous year. Kaplan-Meier method to depict the survival curves and differences in survival were compared using the log-rank tests. Multivariate Cox regression analyses were performed to determine the independent prognostic factors associated with OCSS and OS. IBM SPSS version 22.0 (IBM Corp., Armonk, NY, USA) was also used in the analysis. We used a significance level of P < 0.05, and all tests were two-tailed.

## Results

### Patient characteristic

A total of 4257 OCCC patients were included between 2000 and 2015 in this study (**Table 1**). Of these patients, 1965 (46.2%) and 2292 (53.8%) were diagnosed in 2000-2007 and 2008-2015, respectively. A total of 3334 (78.3%), 167 (3.9%), and 690 (16.2%) patients were White, Black and Asian Americans, respectively. Patients with Asian Americans (P<0.001) or poorly/undifferentiated (P<0.001) were more likely to be diagnosed in later years. Moreover, the number of patients diagnosed with regional stage gradually increases over time, while those diagnosed with localized and distant stage gradually decrease over time (P<0.001). Regarding treatment, 3214 (73.4%) patients were treated with chemotherapy and the number of patients receiving chemotherapy gradually increased over time (P<0.001). A similar distribution of age (P=0.349) or CA125 level before treatment (P=0.107) were found over the study period. A total of 2524 patients were available data for CA125 status, including 1865 (73.9%) who had CA125 ≥35ug/ml. There were 520 (62.7%), 719 (70.5%), and 626 (92.9%) patients who had CA125 ≥35ug/ml in localized, regional, and distant stage diseases, respectively (P<0.001).

**Table 1:** Descriptive demographic and clinical characteristics of patients according to year of diagnosis (n=4257).

Variables	n	2000-2007 (%)	2008-2015 (%)	P
Age (years)				
<65	3311	1541 (78.4)	1770 (77.2)	0.349
≥65	946	424 (21.6)	522 (22.8)	
Race				
White	3334	1603 (81.6)	1731 (75.5)	<0.001
Black	167	64 (3.3)	103 (4.5)	
Asian	690	272 (13.8)	418 (18.2)	
Other	66	26 (1.3)	40 (1.7)	
Grade				
Well differentiated	53	29 (2.7)	24 (1.5)	<0.001
Moderately differentiated	377	214 (19.9)	163 (10.4)	
Poorly/undifferentiated	2218	834 (77.4)	1384 (88.1)	
Unknown	1609	−	−	
SEER stage				
Localized	1521	727 (37.0)	794 (34.6)	<0.001
Regional	1630	671 (34.1)	959 (41.8)	
Distant	1106	567 (28.9)	539 (23.5)	
CA125 level (ug/ml)				
<35	659	188 (24.0)	471 (27.1)	0.107
≥35	1865	595 (76.0)	1270 (72.9)	
Unknown	1733	−	−	
Treatment				
Surgery	1133	631 (32.1)	502 (21.9)	<0.001
Surgery + chemotherapy	3124	1334 (67.9)	1790 (78.1)	

SEER, Surveillance, Epidemiology, and End Results. '-' Indicates as none available.

### Prognostic analysis

The median follow-up was 67 months (range, 0-227 months). The results of the multivariate Cox regression analyses showed that the year of diagnosis was the independent prognostic factor associated with OCSS and had a borderline effect on OS (**Table 2**). Those diagnosed between 2008-2015 had a significantly higher OCSS (hazard ratio [HR] 0.846, 95% confidence interval [CI] 0.754-0.949, P=0.004) compared to those diagnosed between 2000-2007. Similar OS was found between those diagnosed between 2008-2015 and 2000-2007 (HR 0.905, 95%CI 0.816-1.004, P=0.060). Age, race, SEER stage, CA125 status, and chemotherapy receipt were also the independent prognostic factors associated with survival outcomes (**Table 2**).

**Table 2:** Multivariate Cox regression analyses of the independent prognostic factors associated with ovarian cancer-specific survival and overall survival.

Variables	OCSS			OS		
	HR	95%CI	P	HR	95%CI	P
Age (years)						
<65	1			1		
≥65	1.066	1.002-1.134	0.045	1.269	1.206-1.335	<0.001
Race						
White	1			1		
Black	1.422	1.131-1.798	0.003	1.464	1.193-1.797	<0.001
Asian and other races	0.855	0.736-0.993	0.040	0.885	0.776-1.009	0.067
Grade						
Well differentiated	1			1		
Moderately differentiated	1.137	0.638-2.027	0.663	1.159	0.709-1.894	0.556
Poorly/undifferentiated	1.198	0.691-2.075	0.520	1.207	0.756-1.926	0.431
Unknown	1.193	0.687-2.071	0.531	1.238	0.774-1.978	0.373
SEER stage						
Localized	1			1		
Regional	2.140	1.799-2.545	<0.001	1.673	1.463-1.914	<0.001
Distant	10.234	8.697-12.043	<0.001	6.888	6.049-7.843	<0.001
CA125 level (ug/ml)						
<35	1			1		
≥35	1.561	1.273-1.915	<0.001	1.508	1.268-1.794	<0.001
Unknown	1.252	1.013-1.548	0.038	1.275	1.065-1.525	0.008
Treatment						
Surgery	1			1		
Surgery + chemotherapy	0.921	0.805-1.054	0.231	0.817	0.732-0.913	<0.001
Years of diagnosis						
2000-2007	1			1		
2008-2015	0.846	0.754-0.949	0.004	0.905	0.816-1.004	0.060

SEER, Surveillance, Epidemiology, and End Results; OCSS, ovarian cancer-specific survival; OS, overall survival; HR, hazard ratio; CI, confidence interval.

### Survival trends of OCCC from 2000 to 2015

**Figure 2:**
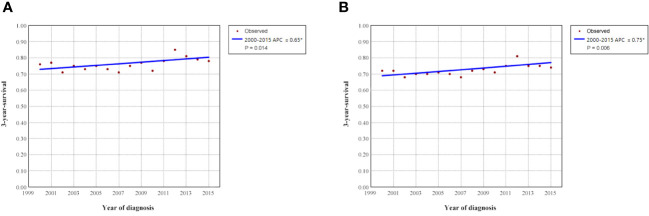
Annual percent change (APC) in 3-year ovarian cancer-specific survival **(A)** and overall survival **(B)** from 2000 to 2015.

**Figure 3:**
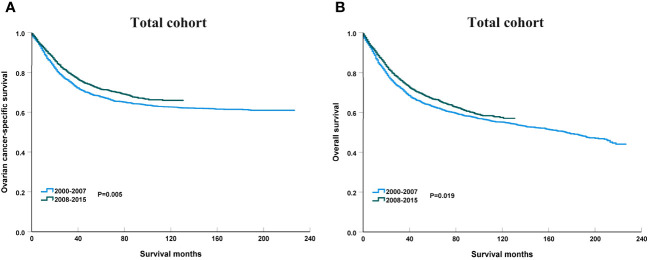
The impact of the years of diagnosis on ovarian cancer-specific survival **(A)** and overall survival **(B)** in the entire cohort.

To clarify the trend in survival of OCCC patients during the study period, we counted the trends of 3-year OCSS and 3-year OS of OCCC patients from 2000 to 2015. The 3-year OCSS rate for patients increased slightly from 2000 (3-year OCSS 76%) to 2015 (3-year OCSS 78%), with an APC value of 0.65 (P=0.014). The trend in 3-year OS was more significant than the change in OCSS over the study period (3-year OS 72% in 2000 and 74% in 2015), with an APC value of 0.75 (P=0.006). **Figure 2** shows the APC in 3-year OCSS and OS over the study period. The survival curves between those diagnosed between 2000-2007 and 2008-2015 have listed in **Figure 3**, which also showed a better OCSS and OS in those diagnosed in later years.

### Survival trends according to SEER staging from 2000 to 2015

**Figure 4:**
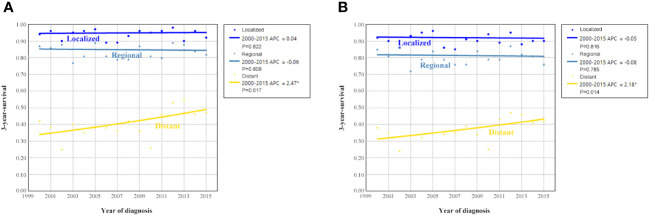
Annual percent change (APC) in 3-year ovarian cancer-specific survival **(A)** and overall survival **(B)** according to SEER staging from 2000 to 2015.

**Figure 5:**
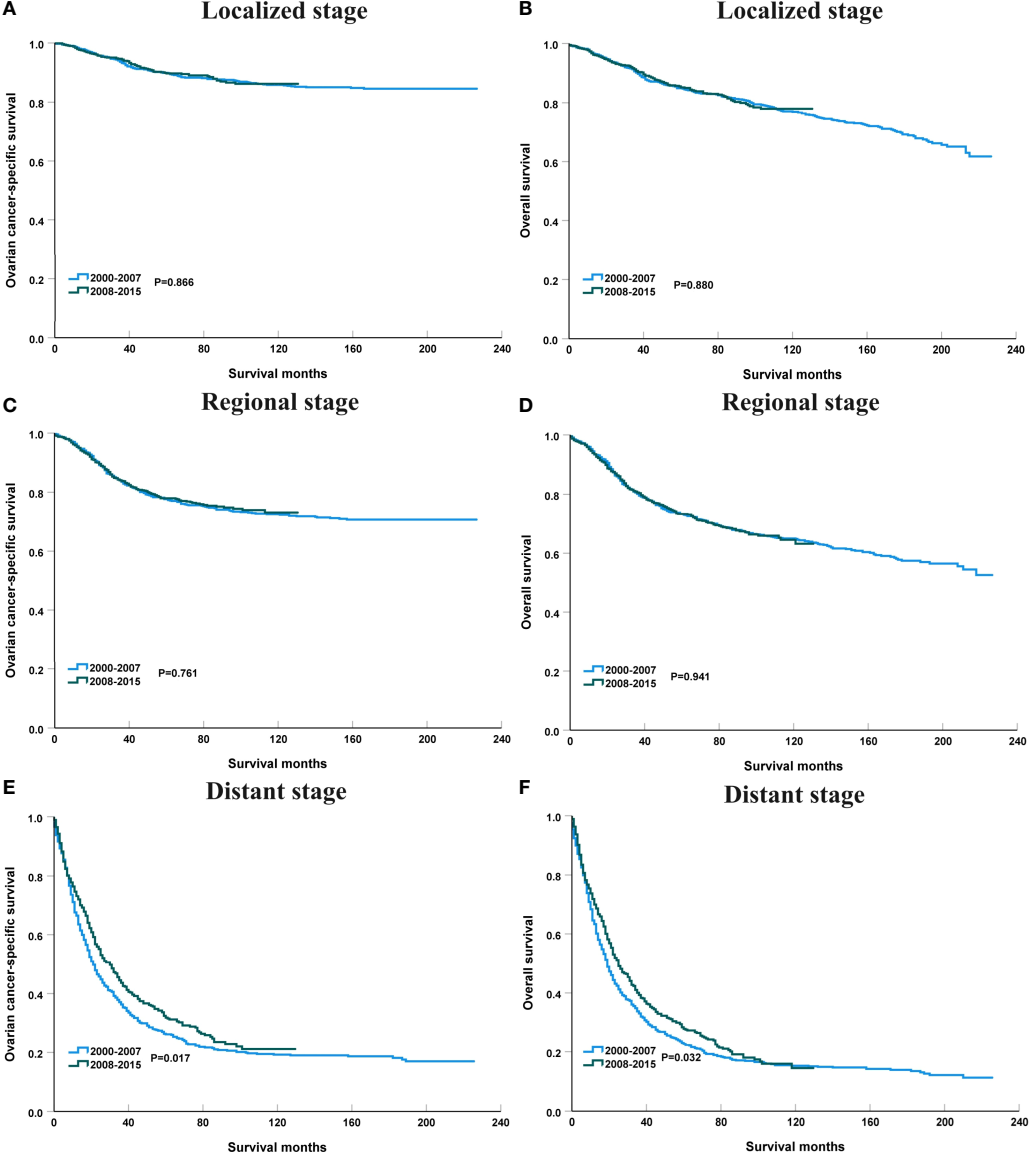
The impact of the years of diagnosis on ovarian cancer-specific survival and overall survival in patients with localized [**(A)**, ovarian cancer-specific survival; **(B)**, overall survival], regional [**(C)**, ovarian cancer-specific survival; **(D)**, overall survival], and distant stage [**(E)**, ovarian cancer-specific survival; **(F)**, overall survival].

**Figure 4** shows the survival trends according to the SEER staging of the OCCC. The survival of patients with distant stage showed a significant trend toward increased, with an APC value of 2.47 in OCSS (3-year OCSS 42% in 2000 and 47% in 2015) (P=0.017) and an APC value of 2.18 in OS (3-year OS 37% in 2000 and 42% in 2015) (P=0.014). However, no significant trends were found in the survival of patients with localized or regional stage diseases. The survival curves between those diagnosed between 2000-2007 and 2008-2015 after stratification by SEER staging have listed in **Figure 5**. Regarding distant stage, those diagnosed between 2008-2015 had a significantly better OCSS (P=0.017) and OS (P=0.032) compared to those diagnosed between 2000-2007. However, similar OCSS and OS were found between those diagnosed between 2000-2007 and 2008-2015 in the localized or regional stage diseases. Similar findings were observed using multivariate Cox regression analyses (**Table 3**).

**Table 3:** Sensitivity analyses of the impact of the year of diagnosis on ovarian cancer-specific survival and overall survival.

Variables	OCSS			OS		
	HR	95%CI	P	HR	95%CI	P
Localized stage						
2008-2015 vs. 2000-2007	0.947	0.688-1.302	0.737	0.990	0.771-1.270	0.935
Regional stage						
2008-2015 vs. 2000-2007	0.908	0.729-1.132	0.390	0.986	0.813-1.196	0.887
Distant stage						
2008-2015 vs. 2000-2007	0.823	0.707-0.958	0.012	0.851	0.737-0.982	0.027
Aged <65 years						
2008-2015 vs. 2000-2007	0.854	0.749-0.974	0.019	0.882	0.781-0.996	0.043
Aged <65 years (localized stage)						
2008-2015 vs. 2000-2007	0.931	0.640-1.352	0.706	0.975	0.709-1.339	0.874
Aged <65 years (regional stage)						
2008-2015 vs. 2000-2007	0.951	0.743-1.216	0.688	0.977	0.781-1.220	0.835
Aged <65 years (distant stage)						
2008-2015 vs. 2000-2007	0.809	0.680-0.962	0.017	0.832	0.705-0.981	0.029
Aged ≥65 years						
2008-2015 vs. 2000-2007	0.858	0.672-1.095	0.217	0.977	0.800-1.193	0.819
Aged ≥65 years (localized stage)						
2008-2015 vs. 2000-2007	1.063	0.576-1.960	0.846	1.029	0.687-1.542	0.888
Aged ≥65 years (regional stage)						
2008-2015 vs. 2000-2007	0.772	0.471-1.265	0.304	1.060	0.722-1.557	0.765
Aged ≥65 years (distant stage)						
2008-2015 vs. 2000-2007	0.838	0.609-1.153	0.279	0.903	0.675-1.209	0.493
Surgery alone						
2008-2015 vs. 2000-2007	1.074	0.836-1.380	0.575	1.182	0.962-1.453	0.112
Surgery alone (localized stage)						
2008-2015 vs. 2000-2007	1.125	0.660-1.918	0.664	1.308	0.899-1.902	0.160
Surgery alone (regional stage)						
2008-2015 vs. 2000-2007	0.995	0.624-1.589	0.985	1.162	0.790-1.709	0.444
Surgery alone (distant stage)						
2008-2015 vs. 2000-2007	1.048	0.728-1.509	0.800	1.051	0.754-1.466	0.768
Surgery + chemotherapy						
2008-2015 vs. 2000-2007	0.804	0.706-0.917	0.001	0.838	0.743-0.945	0.004
Surgery + chemotherapy (localized stage)						
2008-2015 vs. 2000-2007	0.911	0.612-1.356	0.645	0.843	0.604-1.175	0.313
Surgery + chemotherapy (regional stage)						
2008-2015 vs. 2000-2007	0.883	0.690-1.131	0.324	0.936	0.751-1.166	0.554
Surgery + chemotherapy (distant stage)						
2008-2015 vs. 2000-2007	0.782	0.660-0.926	0.004	0.815	0.694-0.957	0.013

OCSS, ovarian cancer-specific survival; OS, overall survival; HR, hazard ratio; CI, confidence interval.

### Survival trends according to age groups from 2000 to 2015

**Figure 6:**
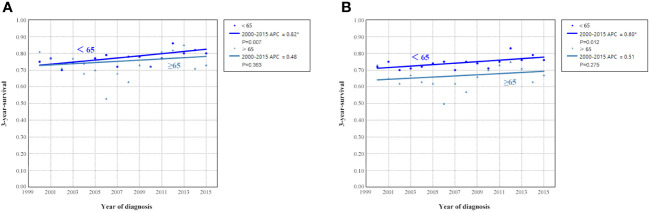
Annual percent change (APC) in 3-year ovarian cancer-specific survival **(A)** and overall survival **(B)** according to age at diagnosis from 2000 to 2015.

**Figure 7:**
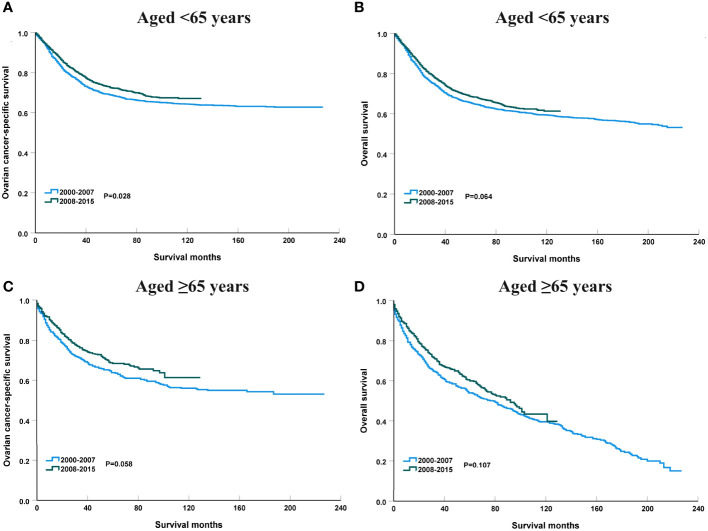
The impact of the years of diagnosis on ovarian cancer-specific survival and overall survival in patients aged <65 years [**(A)**, ovarian cancer-specific survival; **(B)**, overall survival] and those aged ≥65 years [**(C)**, ovarian cancer-specific survival; **(D)**, overall survival].

**Figure 6** shows the APC in 3-year OCSS and OS according to age at diagnosis. Patients aged <65 years showed a significant increase in survival from 2000 to 2015, with an APC value of 0.82 for OCSS (3-year OCSS 75% in 2000 and 80% in 2015) (P=0.007) and an APC value of 0.60 for OS (3-year OS 72% in 2000 and 76% in 2015) (P=0.012). However, the survival trends could not observed for patients aged ≥65 years. Similar findings were observed using multivariate Cox regression analyses (**Table 3**). The survival curves between those diagnosed between 2000-2007 and 2008-2015 in the aged <65 years and aged ≥65 years groups have listed in **Figure 6**. We found a significant effect on OCSS (P=0.028) and a borderline effect on OS (P=0.064) in those diagnosed between 2008-2015 compared to those diagnosed between 2000-2007 in patients aged <65 years using the Kaplan-Meier analysis (**Figure 7**).

**Figure 8:**
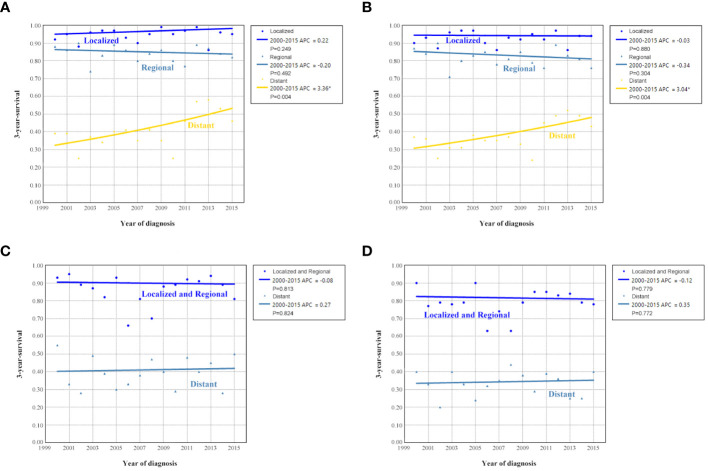
Annual percent change (APC) in 3-year ovarian cancer-specific survival and overall survival according to SEER staging in patients aged <65 years [**(A)**, ovarian cancer-specific survival; **(B)**, overall survival] and aged ≥65 years [**(C)**, ovarian cancer-specific survival; **(D)**, overall survival] from 2000 to 2015.

The sensitivity analyses were performed to investigate the effect of SEER staging on APC according to age at diagnosis. **Figure 7** shows trends in survival in those aged <65 years according to the SEER staging. The significant increase in survival for patients aged <65 years was largely due to the increase in survival for patients with distant stage (3-year OCSS 39% in 2000 and 46% in 2015, P=0.004; 3-year OS 37% in 2000 and 43% in 2015, P=0.004). With an APC value of 3.36 for 3-year OCSS and an APC value of 3.04 for 3-year OS. However, there was no statistically significant change in the survival rate of patients aged <65 years with localized and regional stage diseases over time. In addition, there was also no statistically significant change in the survival rate of patients aged ≥65 years with localized, regional, or distant stage diseases over time (**Figure 8**). Similar findings were observed using multivariate Cox regression analyses (**Table 3**).

### Survival trends by treatment receipt from 2000 to 2015

**Figure 9:**
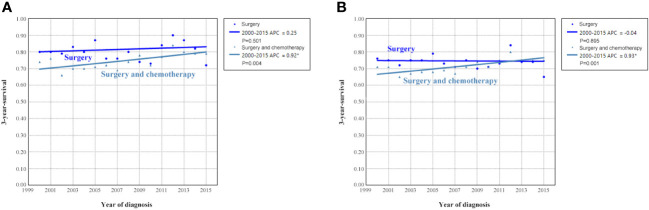
Annual percent change (APC) in 3-year ovarian cancer-specific survival **(A)** and overall survival **(B)** according to treatment receipt from 2000 to 2015.

**Figure 10:**
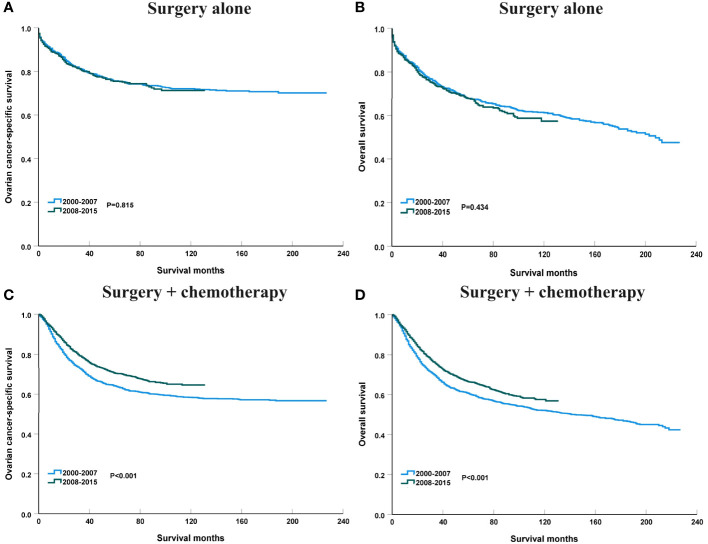
The impact of the years of diagnosis on ovarian cancer-specific survival and overall survival in patients treated with surgery alone [**(A)**, ovarian cancer-specific survival; **(B)**, overall survival] and surgery + chemotherapy [**(C)**, ovarian cancer-specific survival; **(D)**, overall survival].

We analyzed to examine the impact of different treatments on survival rates. Specifically, we focused on patients who underwent surgery alone or a combination of surgery and chemotherapy. **Figure 9** shows the 3-year survival according to treatment. A significant increase in 3-year OCSS was observed for patients treated with surgery combined with chemotherapy, with an APC value of 0.92 (3-year OCSS 74% in 2000 and 79% in 2015) (P=0.004), as well as a trend toward a significant improvement in OS, with an APC value of 0.93 (3-year OS 71% in 2000 and 75% in 2015) (P=0.001), whereas there was no significant change in survival for patients treated with surgery alone. Similar findings were observed using Kaplan-Meier analysis and multivariate Cox regression analyses (**Figure 10** and **Table 3**).

**Figure 11:**
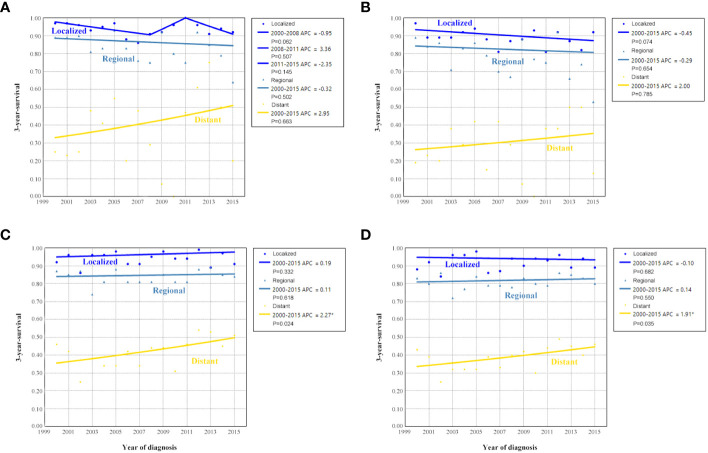
Annual percent change (APC) in 3-year ovarian cancer-specific survival and overall survival according to SEER stage in patients treated with surgery alone [**(A)**, ovarian cancer-specific survival; **(B)**, overall survival] and surgery + chemotherapy [**(C)**, ovarian cancer-specific survival; **(D)**, overall survival].

The sensitivity analyses were performed to investigate the effect of SEER staging on APC according to treatment receipt. The significant increase in survival for patients treated with surgery combined with chemotherapy was largely due to the increase in survival for patients with distant stage (3-year OCSS 46% in 2000 and 51% in 2015, P=0.024; 3-year OS 43% in 2000 and 46% in 2015, P=0.035) (**Figure 10**). In patients with localized or regional stage diseases, there was no statistically significant change in the survival rate of patients who received surgery and chemotherapy over time. Moreover, there was also no statistically significant change in the survival rate of patients with localized, regional, or distant stage diseases over time in those who received surgery alone (**Figure 11**). Similar findings were observed using the multivariate Cox regression analyses (**Table 3**).

## Discussion

OCCC is a distinct type of cancer that has unique features in its occurrence, development, treatment, and prognosis. OCCC has a unique genetic profile with a lower p53 mutation rate (25%) and a lower BRCA1/2 mutation rate (6.3%) but higher mutation rates in ARID1A, PIK3CA, and PTEN compared to high-grade serous EOC. Since inflammatory and epigenetic processes seem to play a predominant role in the pathogenesis of OCCC, immune checkpoint inhibitors, and epigenetic treatment approaches may play an important role in the treatment of these tumor entities (12). In the past, it has not received much attention due to its rarity. However, in recent years, there has been increased interest in researching OCCC, primarily because of its specific clinical characteristics and the varying survival rates observed in early and late-stage patients. In this study, we utilized the SEER database to analyze data of OCCC patients between 2000 and 2015. We aimed to identify any changes in survival trends among OCCC patients over the past decade and explore the influence of different factors on these trends. Our study will enhance the understanding of the disease and provide valuable insights and evidence for future research on treatment modalities.

In this study, we found an increasing trend in the number of OCCC diagnoses between 2000 and 2015 in Asian Americans. Several studies have found that the incidence rate of OCCC in the Asian population is higher than that in the US (22, 23). We should note that those Asian Americans were first-generation immigrants or descendants of immigrants. A previous study conducted in the US identified an increased risk of OCCC among individuals of Asian Pacific Islander ethnicity. However, the study also found that the risk did not significantly vary based on place of birth, indicating that factors such as acculturation or environmental exposure may not strongly influence the association (24). These findings imply that the development of OCCC involves a complex interplay of external and intrinsic factors. The elevated risk observed in Asian Americans may be attributed to genetic predisposition, making it more difficult to modify or mitigate.

Considerable efforts have been made to implement screening programs for early diagnosis of EOC in the general population, but currently, there is no approved strategy (25). This is also reflected economically and cost-effective strategies for early detection and prevention of ovarian cancer have been investigated over the last decade. The cost of treatment per patient with ovarian cancer remains the highest among all cancer types. As an example, the average initial cost in the first year can amount to around US dollar 80,000, whereas the final year cost may increase to US dollar 100,000 (26). The combination of CA125 and transvaginal ultrasound has been explored, but there is limited evidence demonstrating its effectiveness in reducing EOC mortality (27). The number of asymptomatic ovarian masses has increased with the use of prenatal ultrasonography. Among ovarian tumors that complicate pregnancies, approximately 5% are malignant. Currently, surgical intervention is indicated for an ovarian mass over 6 cm in diameter or when symptomatic (28). A recent study also did not support effective screening in average-risk women (29). In our study, we found that approximately 70% of patients had an elevation of CA125, and patients with advanced stage had a higher risk of elevation of CA125, which was similar to the previous studies (30, 31). However, we found a downward trend in patients with distant stage and an upward trend in regional stage. In addition, the overall trend of patients in localized stage was decreasing. There is currently no effective screening strategy for OCCC. Several studies have indicated that the rise in the proportion of OCCC is attributed to increased estrogen exposure and the subsequent rise in rates of endometriosis (9–11). Therefore, further exploration should be conducted to determine whether screening for long-term estrogen exposure and patients with endometriosis can further improve the early diagnosis of OCCC.

Adjuvant chemotherapy using carboplatin and paclitaxel is currently recommended for those with stage IC2 and above (32). However, the role of adjuvant chemotherapy in patients with stage IA to IC disease remains uncertain. The consensus from the European Society for Medical Oncology-European Society of Gynaecological Oncology indicates that adjuvant chemotherapy is not recommended for stage IA, IB, or IC1 OCCC with complete surgical staging (33). A recent SEER study showed that there was no OS benefit for patients with stage IC OCCC receiving adjuvant chemotherapy (5-year OS, 83% vs. 80%, P=0.62) (34). Several small sample studies also found that chemotherapy did not improve the survival of stage I-II OCCC (35, 36). Moreover, a previous study conducted at two tertiary centers in Toronto showed a potential benefit of adjuvant chemotherapy in reducing disease recurrence, although this did not result in an improved OS in stage I-II OCCC (37). In our study, we observed that chemotherapy did not enhance the survival of patients in the localized and regional stages, but the use of chemotherapy improved the survival of patients with distant stage. However, a cohort study conducted using the National Cancer Database demonstrated a benefit in OS for patients with stage I OCCC who received adjuvant chemotherapy (38). Considering the limited conclusive evidence regarding its efficacy in this specific subgroup, the decision to proceed with adjuvant chemotherapy or opt for observation should be personalized after thorough patient counseling.

Several studies have shown that OCCC is considered to be relatively insensitive to chemotherapy compared to other subtypes of EOC. In a study of 27 patients with stage III/IV OCCC and residual disease after surgery, the response rate to platinum-based chemotherapy was only 11.1% (39). Additionally, the response rate to chemotherapy for OCCC patients with recurrent disease was reported to be as low as 6-8% (40). A previous study has found a high probability of ARID1A gene mutation in OCCC (49%), and there is a significant correlation between ARID1A gene mutation and platinum resistance of patients (10). There is also a relationship with the specific tumor microenvironment of OCCC (41–43). Our study found that patients with distant stage receiving surgery and chemotherapy had survival improvement over the years, which may be related to the improvement of chemotherapy regimen methods and exploration of targeted drugs in patients with distant stage OCCC, including the use of bevacizumab in distant stage OCCC. Bevacizumab was approved for the treatment of EOC starting in 2007 and several studies have found that the use of bevacizumab was associated with a higher response rate and better survival outcomes in relapsed or metastatic OCCC (17–19, 44). New chemotherapy regimens, including docetaxel and irinotecan (45), and gemcitabine (46, 47), may improve the treatment sensitivity of platinum-resistant patients. Moreover, the advent of new targeted therapies may further improve patient survival in the future (48).

While OCCC is not as chemosensitive as the more common high-grade serous EOC, there is very limited data regarding the actual clinical benefit of chemotherapy in OCCC patients. Therefore, it is crucial to emphasize the need for novel targeted treatments for the management of OCCC. Several studies have found that OCCC had promising responses to immune checkpoint inhibitors (49–51). Moreover, the combination of immune checkpoint inhibitors and targeting angiogenesis including bevacizumab or lenvatinib also showed clinical benefit in OCCC (52–54). Notch and VEGF are essential in ovarian cancer angiogenesis and Notch has also been related to chemoresistance. Thus, Notch targeting, and mainly dual targeting of Notch and VEGF, is a promising strategy in ovarian cancer. The combination of Notch inhibition with chemotherapy or antiangiogenics showed interesting activity in early-phase clinical studies. Navicixizumab, a dual anti-Dll4 and anti-VEGF in combination with weekly paclitaxel showed a response rate of 43% in heavily pretreated platinum-resistant patients (55). However, we need to note that the survival improvement is not very significant, and the CSS and OS of distant stage patients indicate an improvement of 5% and 5% between 2000 and 2015, respectively. In addition, we should also note that studies on multiple innovative drugs, including cabozantinib (56), temsirolimus (57), and ENMD-2076, did not significantly improve patient survival (58). Therefore, further exploration based on molecular stratification should be needed in the future to optimize treatment strategies for OCCC patients.

Age itself is a poor prognostic factor in patients with EOC (59). Our study also showed better OCSS and OS in those aged <65 years compared to those aged ≥65 years. Our results also demonstrated a significant survival improvement in patients aged <65 years, especially for patients with distant stage. For young patients, there has been little overall change in the survival rates for localized and regional stage diseases over the years, which may be correlated with the overall stability in treatment patterns among these patients over the past years. However, in patients with distant stage, it is possible that more of them have been enrolled in clinical trials for new drugs or have received more aggressive treatments. In those aged ≥65 years, we found no survival improvement over the years, including those with distant stages. The reasons are not fully clarified. Several factors could contribute to the survival difference by different age groups, including comorbidity, more advanced stage at diagnosis, toxic effects of chemotherapy, or that elderly patients are less often treated with optimal surgery or chemotherapy (60). Moreover, in other histotypes of EOC, age is associated with differences in underlying biology. Therefore, there may also be different biological behaviors exhibited among age groups in OCCC. Further studies are needed to investigate the disparities in biological behaviors among age groups in OCCC (61–64). Finally, most clinical trials exclude elderly individuals or have a median age of only around 60 years (65–67). Due to the potential survival benefits inherent in participating in various clinical trials (68), suitable elderly populations should also participate in clinical trials to evaluate the impact of new treatment regimens on patient survival outcomes as much as possible.

Some limitations should be mentioned. First, the retrospective nature of the study, the long duration of the study period, and the use of different therapeutic approaches are inherent biases in the research design. Second, the lack of a centralized pathology review may have resulted in some misclassification of the histological types. High-grade serous EOC with clear cell change has historically been frequently misclassified as OCCC, which would account for some of the trends observed in the study, such as the decrease in distant stage disease diagnoses (69). Third, the SEER database did not record information regarding chemotherapy regimens, chemotherapy cycles, chemotherapy completion rates, targeted therapy, etc. Fourth, information about comorbidities was also not recorded in the SEER database, which might cause bias in the results. Moreover, some of the findings that showed borderline or marginal significance may benefit from long-term follow-up in order to enhance the statistical power. Finally, adjustment for multiple testing was not performed for this study.

## Conclusions

In conclusion, our study observed a significant increase in the survival outcomes in OCCC from 2000 to 2015, and patients aged <65 years and those with distant stage experienced a greater improvement in survival.

## Data availability statement

The raw data supporting the conclusions of this article will be made available by the authors, without undue reservation.

## Ethics statement

As the SEER database consists of de-identified information, the study was exempt from the approval process of the Institutional Review Boards of the First Affiliated Hospital of Xiamen University. The studies were conducted in accordance with the local legislation and institutional requirements.

## Author contributions

B-QT: Conceptualization, Data curation, Writing – original draft. S-WW: Conceptualization, Data curation, Formal analysis, Writing – original draft. J-YX: Conceptualization, Data curation, Investigation, Methodology, Writing – review & editing. S-GW: Funding acquisition, Resources, Validation, Visualization, Writing – review & editing. JZ: Conceptualization, Data curation, Investigation, Resources, Visualization, Writing – review & editing.
